# Supplementary figures and images for: TweepFake: About detecting deepfake tweets
Source: PLoS One. 2021 May 13;16(5):e0251415. doi: 10.1371/journal.pone.0251415 (PMC8118345; doi:10.1371/journal.pone.0251415)

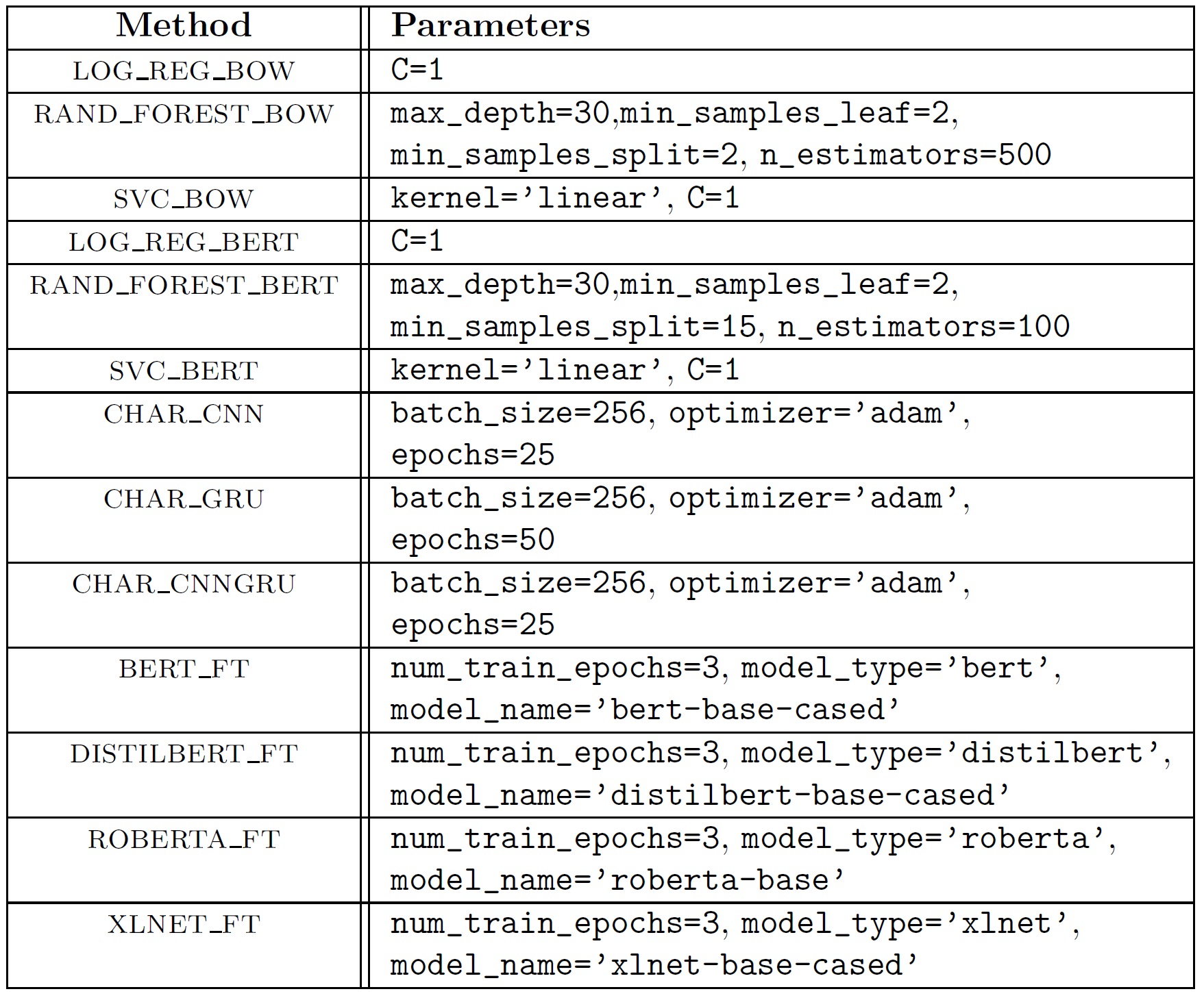

Supplement: S1 Table — Parameter values used in the final experimentation on the test set. (TIFF) [file pone.0251415.s001.tiff]

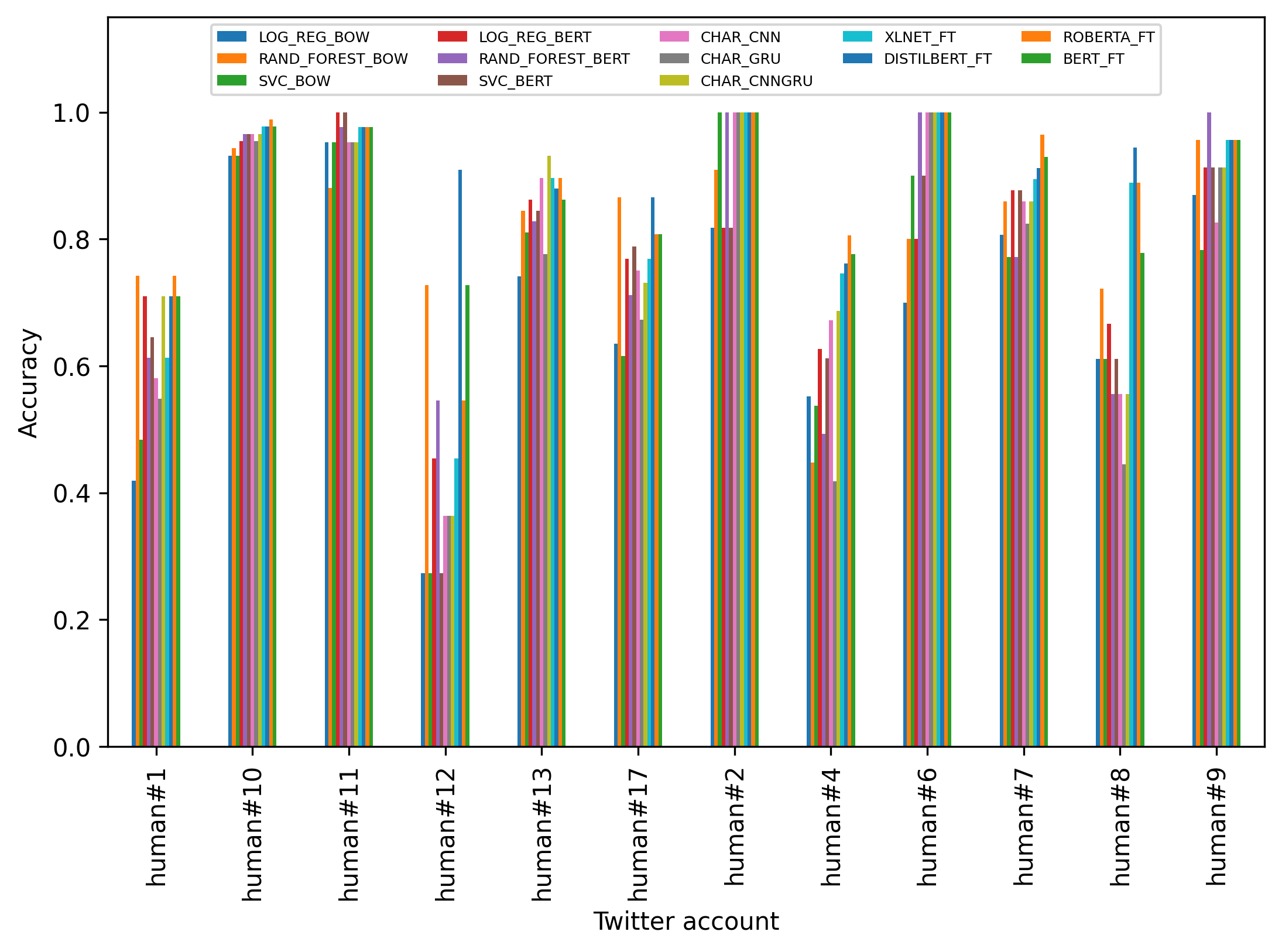

Supplement: S1 Fig — Detection Accuracy of tested methods on ‘human’ accounts with at least 10 examples. (TIF) [file pone.0251415.s002.tif]

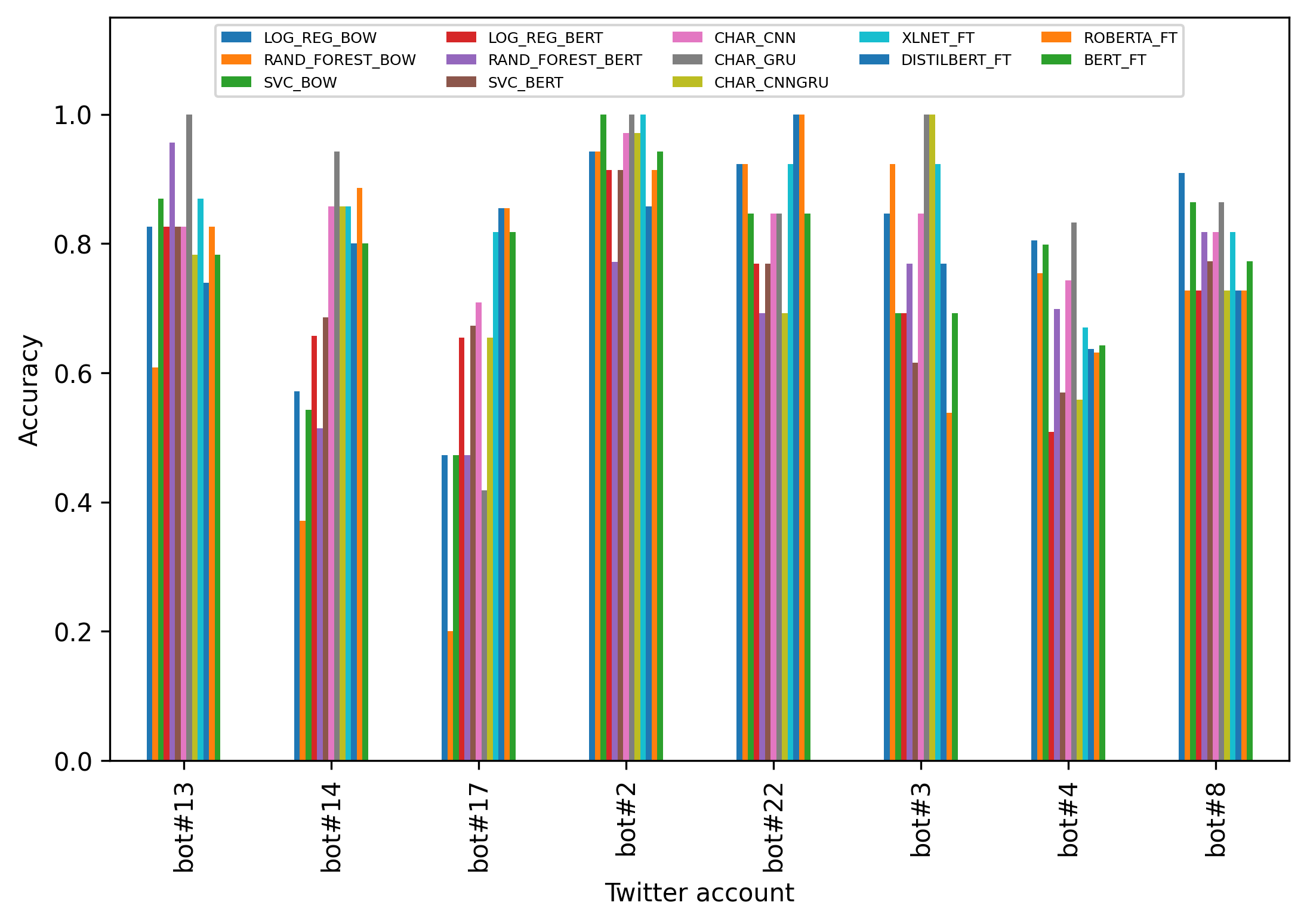

Supplement: S2 Fig — Detection Accuracy of tested methods on ‘gpt2’ accounts with at least 10 examples. (TIF) [file pone.0251415.s003.tif]

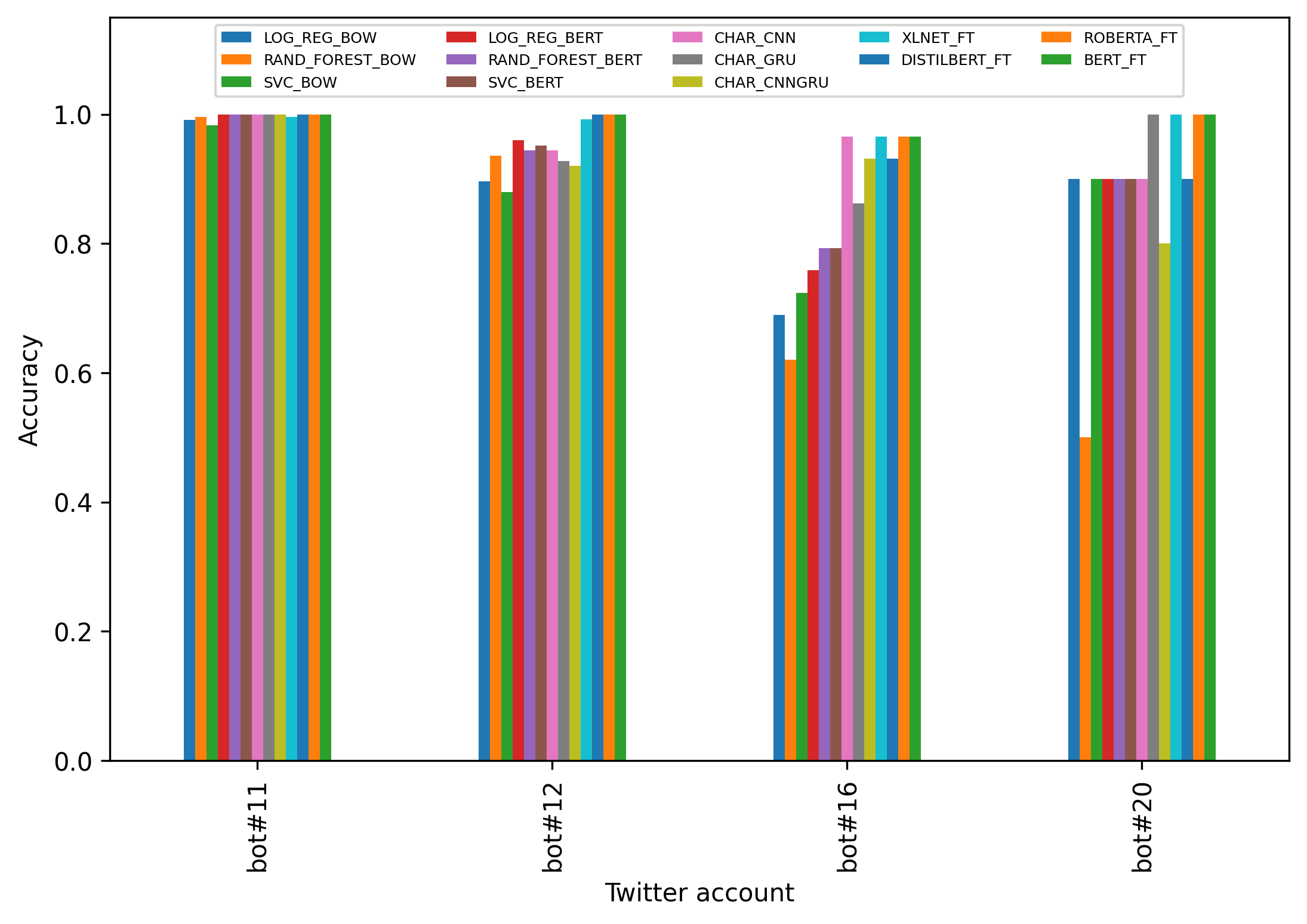

Supplement: S3 Fig — Detection Accuracy of tested methods on ‘rnn’ accounts with at least 5 examples. (TIF) [file pone.0251415.s004.tif]

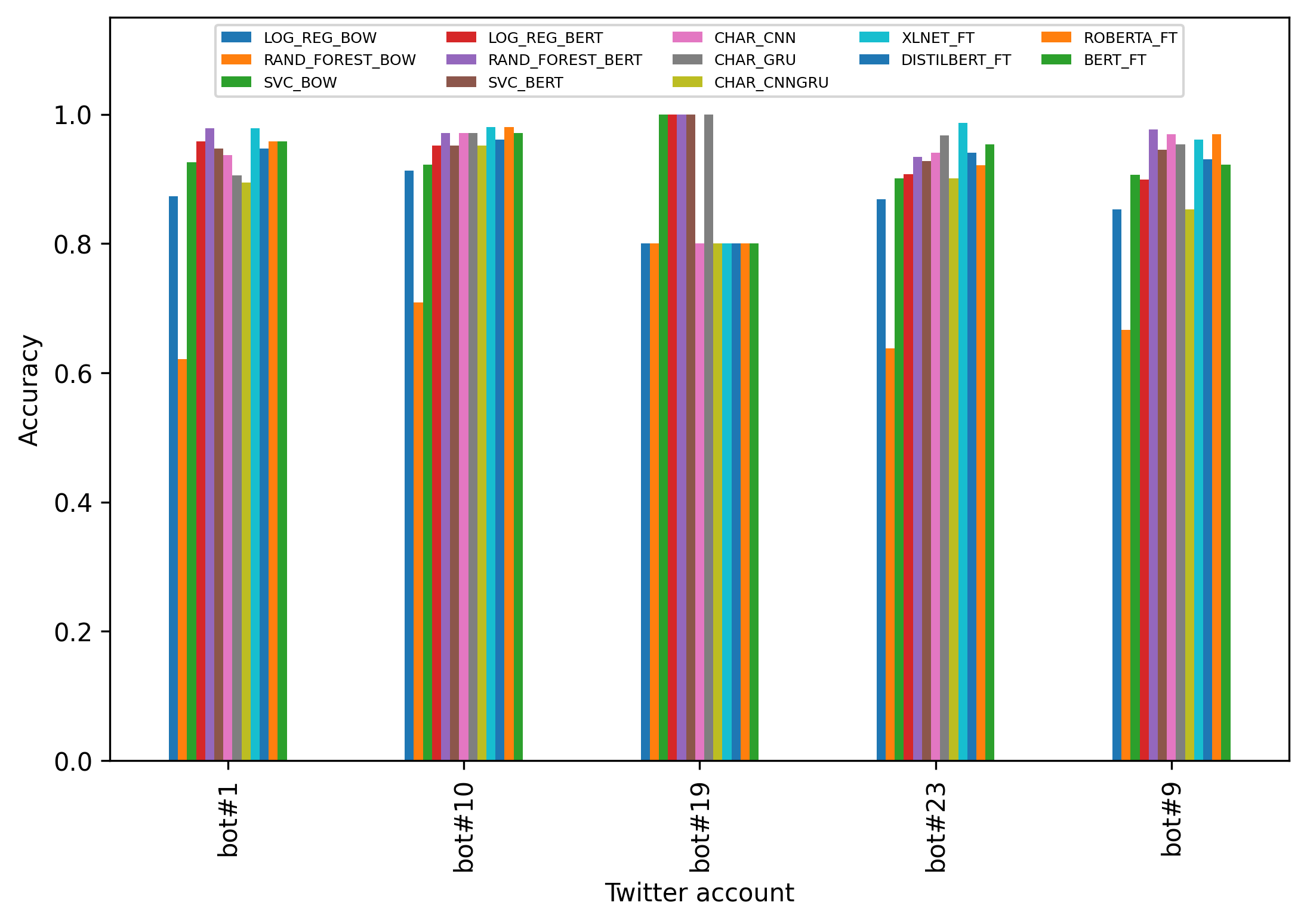

Supplement: S4 Fig — Detection Accuracy of tested methods on ‘others’ accounts with at least 5 examples. (TIF) [file pone.0251415.s005.tif]
